# The Tyrosine-Autokinase UbK Is Required for Proper Cell Growth and Cell Morphology of *Streptococcus pneumoniae*

**DOI:** 10.3389/fmicb.2019.01942

**Published:** 2019-09-03

**Authors:** Anaïs Pelletier, Céline Freton, Clément Gallay, Jennyfer Trouve, Caroline Cluzel, Mirita Franz-Wachtel, Boris Macek, Jean-Michel Jault, Christophe Grangeasse, Sébastien Guiral

**Affiliations:** ^1^Molecular Microbiology and Structural Biochemistry, UMR 5086 CNRS/Université Lyon 1, Lyon, France; ^2^Laboratoire de Biologie Tissulaire et d’Ingénierie Thérapeutique, UMR 5305 CNRS/Université Lyon 1, Lyon, France; ^3^Proteome Center Tübingen, University of Tübingen, Tübingen, Germany

**Keywords:** *Streptococcus pneumoniae*, protein phosphorylation, tyrosine-kinase, ATP hydrolysis, cell-morphogenesis

## Abstract

Protein phosphorylation is a key post-translational modification required for many cellular functions of the bacterial cell. Recently, we identified a new protein-kinase, named UbK, in *Bacillus subtilis* that belongs to a new family of protein-kinases widespread in bacteria. In this study, we analyze the function of UbK in *Streptococcus pneumoniae*. We show that UbK displays a tyrosine-kinase activity and autophosphorylates on a unique tyrosine *in vivo*. To get insights into its cellular role, we constructed a set of pneumococcal *ubk* mutants. Using conventional and electron microscopy, we show that the *ubk* deficient strain, as well as an *ubk* catalytic dead mutant, display both severe cell-growth and cell-morphology defects. The same defects are observed with a mutant mimicking permanent phosphorylation of UbK whereas they are not detected for a mutant mimicking defective autophosphorylation of UbK. Moreover, we find that UbK phosphorylation promotes its ability to hydrolyze ATP. These observations show that the hydrolysis of ATP by UbK serves not only for its autophosphorylation but also for a distinct purpose essential for the optimal cell growth and cell-morphogenesis of the pneumococcus. We thus propose a model in which the autophosphorylation/dephosphorylation of UbK regulates its cellular function through a negative feedback loop.

## Introduction

*Streptococcus pneumoniae* (the pneumococcus) is a Gram-positive bacterium, living as a commensal in healthy adults and children. In immature and/or immunocompromised people, the pneumococcus can, however, become pathogenic and causes diseases that range from otitis, pneumonia to meningitis with sepsis (Kadioglu et al., 2008; Henriques-Normark and Normark, 2010). Despite the availability of antibiotic treatments and vaccines, *S. pneumoniae* still kills more than 1.2 million persons each year and is in the WHO list of priority pathogens for research and development of new antibiotics (Tacconelli, 2017).

Evidences have accumulated that protein phosphorylation on hydroxylated residues (i.e., serine, threonine and tyrosine) catalyzed by serine/threonine-kinases and tyrosine-kinases is crucial for the biology of bacteria (Manuse et al., 2016; Mijakovic et al., 2016). eSTKs (for eukaryotic-like Serine and Threonine kinases), that possess a catalytic domain structurally homologous to that of eukaryotic protein-kinases, have been shown to regulate different physiological processes like the cell cycle, virulence and central and secondary metabolisms (Burnside and Rajagopal, 2012; Mijakovic and Macek, 2012; Canova and Molle, 2014; Fleurie et al., 2014b; Wright and Ulijasz, 2014; Dworkin, 2015; Manuse et al., 2016). eSTKs are widespread in bacteria, but with a highly variable distribution (Dworkin, 2015). On the other hand, phosphorylation on tyrosine is mainly achieved by the bacterial idiosyncratic protein-tyrosine kinase family BY-kinases (Bacterial tyrosine kinases) (Grangeasse et al., 2007; Jadeau et al., 2008; Mijakovic et al., 2016). Like eSTKs, BY-kinases regulate several biological processes and their best studied function concerns their role in the biosynthesis and export of extracellular polysaccharides (Standish et al., 2014; Nourikyan et al., 2015; Mijakovic et al., 2016). They are also widely conserved in bacterial genomes and most of bacterial species encode for at least one BY-kinase (Jadeau et al., 2012).

In the pneumococcus, only one eSTK and one BY-kinase, StkP and D respectively, are produced. Recent studies have demonstrated the critical role of D in the polysaccharide capsule synthesis and export as well as the coordination of this process with the cell cycle (Henriques et al., 2011; Nourikyan et al., 2015; Mercy et al., 2019). The capsule is the main virulence factor of the pneumococcus and its composition is highly variable (more than 90 serotypes known to date). On the other hand, StkP is the central regulator of cell division and morphogenesis (Beilharz et al., 2012; Fleurie et al., 2012, 2014a,b; Grangeasse, 2016; Zucchini et al., 2018).

We have recently identified an unprecedented type of protein-kinase in *Bacillus subtilis* (Nguyen et al., 2017). This protein was named UbK for Ubiquitous bacterial Kinase as it is present in most bacterial genomes. Strikingly, *ubk* genes are found neither in *Archea* nor in eukaryotic genomes (Teplyakov et al., 2002). UbK proteins possess the canonical Walker A-motif G/AX4GKT/S found in the large family of the P-loop proteins, including BY-kinases (Leipe et al., 2002; Grangeasse et al., 2007). However, besides this, the crystal structures of UbK from *Haemophilus influenzae* and *B. subtilis* showed that the structure of UbK proteins share little similarities with that of BY-kinases and other ATP-binding proteins with a Walker A motif (Reinstein et al., 1990; Nguyen et al., 2017). Interestingly, UbKs of *H. influenzae*, *E. coli* or *B. subtilis* show a weak ATPase activity (Campbell et al., 2007; Karst et al., 2009) but their ability to autophosphorylate and to phosphorylate *in vitro* the surrogate substrate Myelin-Basic-Protein has been reported only for UbK from *B. subtilis* (Nguyen et al., 2017). In *B. subtilis*, a recent investigation suggests a possible role in resistance to oxidative stress (Nguyen et al., 2017). *ubks* were described as being essential genes in *S. pneumoniae* (Zalacain et al., 2003; Havarstein et al., 2006), *B. subtilis* (Kobayashi et al., 2003), *Mycoplasma pulmonis* (French et al., 2008) and *E. coli* (Freiberg et al., 2001; Campbell et al., 2007) On the other hand, other studies have concluded that *ubk* would be dispensable in *S. pneumoniae* (Molzen et al., 2011), *B. subtilis* (Hunt et al., 2006; Karst et al., 2009; Nguyen et al., 2017), and *Anabaena* sp. strain (Yoon et al., 2003). One can therefore not exclude that these opposing observations either convey the presence of suppressive mutations appearing when deleting *ubk* genes or that the dispensability/essentiality of *ubk* is strain-specific.

Here, we analyze the cellular function of the pneumococcal UbK (*spr1761* in the R6 strain) together with its kinase and ATP hydrolysis activities. We notably demonstrate that UbK ATP hydrolysis activity, but not autophosphorylation, is crucial for *S. pneumoniae* growth. However, we also show that UbK autophosphorylation increases ATP hydrolysis and is detrimental for the cell growth. Last, we observe that cell growth defects correlate with an abnormal cell morphology. Therefore, we propose a model in which autophosphorylation/dephosphorylation of UbK finely tunes and balances its ATP hydrolysis, an activity that is required for the optimal growth and proper morphogenesis of the pneumococcus cell growth. Altogether, this work highlights the importance of this new atypical tyrosine-kinase in the bacterial cell physiology.

## Results

### *ubk* Is Essential for *Streptococcus pneumoniae*

**FIGURE 1:**
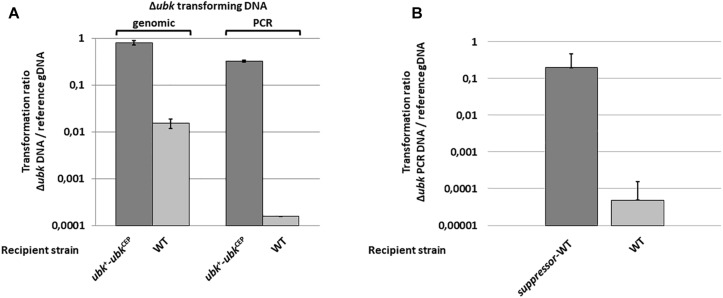
Deletion of *ubk* and co-selection of a suppressive mutation. **(A)** Compared transformation frequencies of the WT (clear bars) and the *ubk*^+^-*ubk*^CEP^ (dark bars) recipient strains transformed either with genomic DNA of the Δ*ubk*:*spc*-*rpsL* original *ubk* mutant (genomic) or with a PCR overlapping the Δ*ubk*:*spc*-*rpsL* mutation (PCR). The transformation ratio Δ*ubk* DNA/reference gDNA represents the Spc^R^ transformants numbered with Δ*ubk* transforming gDNA or Δ*ubk* transforming PCR DNA relative to Spc^R^ transformants numbered with the Δ*phpP*-*stkP*:*spc*-*rpsL* transforming genomic control DNA used as a reference (reference gDNA). **(B)** Compared transformation frequencies of the WT (clear bars) and the *suppressor*-WT (dark bars) recipient strains transformed with a PCR overlapping the Δ*ubk*:*kan*-*rpsL* mutation. The transformation ratio Δ*ubk* PCR DNA/reference gDNA represents the Kan^R^ transformants numbered with the Δ*ubk*:*kan*-*rpsL* transforming PCR DNA relative to Kan^R^ transformants numbered with the Δ*spr1424*:*kan*-*rpsL* transforming genomic control DNA used as a reference (reference gDNA). For **(A,B)** panels, bars represent standard deviations. Experiments were led in triplicate.

To delete *ubk*, we used the Janus markerless and non-polar strategy (Sung et al., 2001). While transformants of the R6 derivative strains were usually obtained after a 16-h culture with transformation levels ranging from 1 to 10%, small colonies of kanamycin resistant mutants were obtained with very low frequency (0.001^%^) after 30 h of growth. We confirmed that *ubk* was properly deleted and substituted for the Janus cassette (Δ*ubk*:*kan-rpsL*) in these mutant colonies by PCR amplification and sequencing of the targeted locus. This observation suggested that *ubk* was either crucial for the pneumococcus biology or that deletion of *ubk* generates a polar effect affecting the expression of downstream genes. To differentiate between the two possibilities, we first expressed *ubk* ectopically using the pCEP maltose-inducible platform (Guiral et al., 2006) in WT cells (*ubk*^+^-*ubk*^CEP^). Then, we substituted the chromosomal copy of *ubk* for the Janus cassette (Δ*ubk*:*kan-rpsL-ubk*^CEP^). Colonies were readily obtained with normal growth and transformation frequency. This excluded the second hypothesis and suggested that the deletion of *ubk* requires suppressive mutations(s). To check this, we statistically analyzed the number of clones obtained by transformation of the WT and the *ubk*^+^-*ubk*^CEP^ strains either with a whole genomic DNA of a Δ*ubk*:*spc-rpsL* mutant or with a Δ*ubk*:*spc-rpsL* PCR amplified DNA fragment (Figure 1A). The *ubk*^+^-*ubk*^CEP^ strain transformed efficiently the two donor DNAs. By contrast, the WT strain transformed the genomic DNA with a 2 log decreased efficiency compared to the *ubk*^+^-*ubk*^CEP^ strain and transformed the PCR DNA with a 4 log decreased efficiency compared to the *ubk*^+^-*ubk*^CEP^ strain. This suggested the presence of a single suppressive mutation co-selected with *ubk* deletion in the genome of original mutants. To identify this suppressive mutation, we performed whole genome sequencing of two independent Δ*ubk* mutants together with that of the WT parental strain. A single mutation differentiating the WT and the Δ*ubk* mutant genomes was unambiguously identified. This mutation consisted in an A to C transversion in position 362 relative to the ATG of the *spr1397* (*asnS*) coding sequence. The mutation led to an aspartate to alanine amino-acid substitution in position 121 of the AsnS protein. *spr1397* was shown already to be an essential gene (Thanassi et al., 2002). We nevertheless transferred the D121A mutation in the *spr1397* gene of WT strain (*asnS-D121A*) and the resulting mutant, called suppressor-WT, was transformed again by the Janus cassette to inactivate chromosomal *ubk*. Colonies were readily obtained with normal transformation level and growth. Confirming that the single A362 to C362 transversion in the *asnS* gene is required to get cells devoid of *ubk* (Figure 1B).

### UbK Autophosphorylates on a Single Tyrosine Residue *in vivo*

**FIGURE 2:**
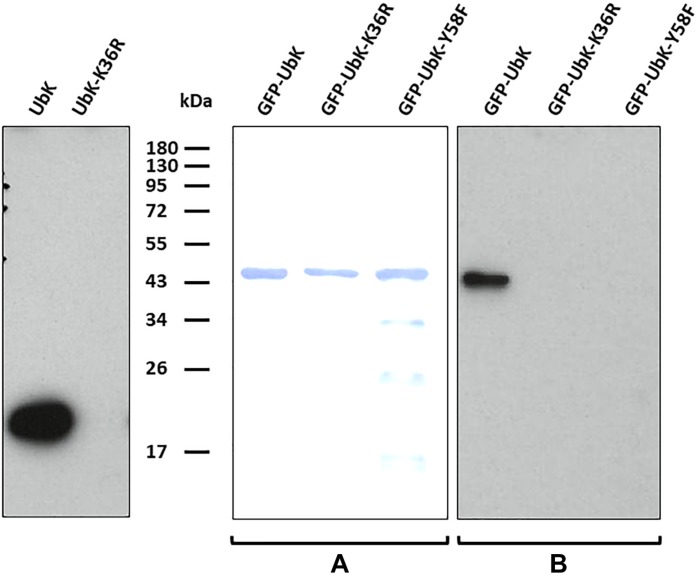
Autophosphorylation of UbK mutant proteins purified from *E. coli* or purified directly from *S. pneumoniae.* **(Left)** Immunodetection with antiphosphotyrosine antibodies of Tyrosine phosphorylation of UbK and UbK-K36R purified from *E. coli*. **(Middle and right)** Immunodetection with antiphosphotyrosine antibodies of Tyrosine-autophosphorylation of GFP-UbK and GFP-UbK mutant proteins directly immuno-purified from *S. pneumoniae*. (A) Staining with Coomassie blue of GFP-UbK, GFP-UbK-K36R and GFP-UbK-Y58F. (B) Immunodetection of Tyrosine-autophosphorylation on Tyr58 of GFP-UbK. In both cases proteins were analyzed by SDS-PAGE, transferred onto a PVDF membrane and immunodetection of Tyrosine phosphorylation was done with antiphosphotyrosine 4G10 antibodies.

UbK of *S. pneumoniae* was previously shown *in vitro* either able to autophosphorylate on tyrosine and to phosphorylate the surrogate substrate myelin basic protein (MBP) (Nguyen et al., 2017). We first tested the relevance of UbK autophosphorylation *in vivo*. For that, we purified UbK fused to the GFP (GFP-UbK) from pneumococcal cells (strain Δ*ubk-gfp-ubk*^CEP^). As control, we also constructed a strain expressing a UbK kinase-dead form in which the conserved lysine 36 of the Walker A motif is mutated for an arginine (strain Δ*ubk-gfp-ubk*K36R^CEP^). Indeed, mutation of this catalytic lysine prevents ATP hydrolysis of Walker A ATP-binding proteins (Reinstein et al., 1990; Soulat et al., 2007; Karst et al., 2009). In addition, we checked that Ubk-K36R overexpressed and purified from *E. coli* cells is unable to autophosphorylate on tyrosine *in vitro* (Figure 2). The same observation was made previously with UbK (i.e., YdiB) from *B. subtilis* mutated on lysine 41 of the Walker A (Nguyen et al., 2017). We then prepared crude extracts of the Δ*ubk-gfp-ubk*^CEP^ and the Δ*ubk-gfp-ubk*K36R^CEP^ pneumococcal strains and performed GFP trap using GFP antibodies immobilized on agarose. Immuno-purified proteins were then analyzed by SDS-PAGE and UbK phosphorylation was analyzed by Western blot using the 4G10 antiphosphotyrosine antibody. As shown in Figure 2, a phosphorylation signal was detected for GFP-UbK but not for GFP-UbK-K36R confirming that UbK is autophosphorylated on tyrosine *in vivo*. Then, we analyzed GFP-UbK by LC-MS/MS mass spectrometry. It showed unambiguously that GFP-UbK was phosphorylated on the tyrosine 58 (Supplementary Figure 1A). To confirm this, and check if Y58 represented the single autophosphorylation site of UbK, we constructed a strain expressing GFP-UbK is which Y58 was substituted for a phenylalanine (strain Δ*ubk-gfp*-*ubk*Y58F^CEP^). Using the same procedure, GFP-UbK-Y58F was GFP-trapped and analyzed by SDS-PAGE and Western blot with the 4G10 antiphosphotyrosine antibody. No phosphorylation signal was detected (Figure 2). Then, we performed Western blots and immunodetections with the antiphosphotyrosine antibodies on whole-cells extracts of WT and Δ*ubk* cells to determine if UbK is responsible for the phosphorylation of pneumococcal endogenous proteins *in vivo* (Supplementary Figure 1B). We did not detect phosphorylation signals in WT cells that are not found in Δ*ubk* cells, suggesting that UbK is unable to phosphorylate a substrate, or only at very low levels undetectable by immunolabelling. The signal detected around 40 kDa in both strains suggested that an unknown protein might be phosphorylated on tyrosine but independently of UbK. In addition, to determine whether UbK autophosphorylates in *cis* or *trans*, we incubated inactive UbK-K36R and UbK with radioactive [α-^32^P]-ATP. The difference in size of the tags fused to UbK-K36R and UbK (Supplementary Table 1) that were used for their purification allowed to separate the two proteins by SDS-PAGE. Autoradiograghy of the gel showed that UbK displayed a radioactive signal whereas UbK-K36R did not (Supplementary Figure 1C). This showed that UbK is unable to *trans*-phosphorylate UbK-K36R and that UbK only *cis*-autophosphorylated. UbK is therefore a tyrosine-autokinase *cis*-autophosphorylating on a single tyrosine, namely tyrosine 58.

### ATP Hydrolysis by UbK but Not UbK Autophosphorylation Is Required for Cell Growth

**FIGURE 3:**
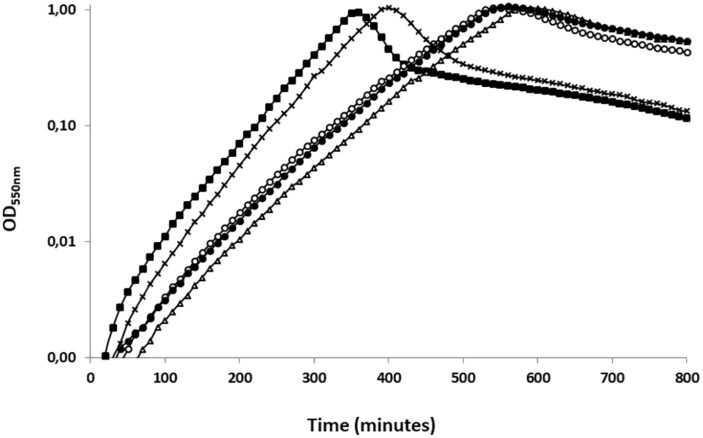
Representative growth of *ubk* mutants. Growth curves of *ubk* mutants at 37°C: WT (black squares), Δ*ubk* (white triangles), *ubk*-K36R (white circles), *ubk*-Y58E (black circles), *ubk*-Y58F (black crosses). Bacteria were diluted so that 2.10^5^ cells were added at *t* = 0 min. *ubk*-Y58F strain grows like the WT strain whereas Δ*ubk*, *ubk*-K36R and *ubk*-Y58E strains display an increased lag phase and a reduced generation time during the exponential growth phase. Growths were led in triplicate.

To assess the function of UbK autophosphorylation, we first analyzed the growth of different *ubk* mutants. As control, we first checked that the suppressive mutation D121A has not effect on the pneumococcal growth (Supplementary Figure 2). Then, we generated strains expressing the different forms of UbK (mutated on the catalytic lysine and Y58) from the *ubk* chromosomal locus under the control of the native promoter. Two strains expressing UbK carrying either a mutation substituting Y58 for a phenylalanine or for a glutamic acid (strains *ubk-*Y58F and *ubk*-Y58E respectively) were constructed. Indeed, mutation of a phosphorylatable tyrosine for a phenylalanine (phospho-ablative) or a glutamic acid (phospho-mimetic) is classically used as the best proxy to mimic a deficient or permanent phosphorylation, respectively (Bidnenko et al., 2013; Lee et al., 2014; Vilcheze et al., 2014). The mutated chromosomal copy represented therefore the only source of UbK protein in these mutant strains (*ubk*-K36R*, ubk*-Y58F, and *ubk*-Y58E*)*. The analysis of the cell growth of these mutants revealed an unexpected behavior. While cells expressing UbK-K36R grew slowly and similarly to Δ*ubk* cells, reflecting that UbK activity is crucial for the pneumococcal growth, *ubk-*Y58E cells also displayed the same slow and delayed growth (Figure 3). These observations implied that the ability of UbK to bind and hydrolyze ATP is not only dedicated to its autophosphorylation but likely serves another purpose required for the growth of the pneumococcus. On the other hand, it also suggested that permanent phosphorylation of UbK is detrimental for the cell. Supporting this, *ubk-*Y58F cell grew properly (Figure 3). Moreover, we constructed two other mutants in which we mutated both the catalytic lysine and the autophosphorylation site, generating thus the *ubk-*K36R-Y58F and the *ubk-*K36R-Y58E strains. We observed that these two strains behaved like the *ubk-*K36R and Δ*ubk* mutants with a slow and delayed growth (Supplementary Figure 3 and Figure 3). The *ubk-*K36R mutation is therefore epistatic over *ubk* phosphorylation and ATP hydrolysis is predominant for the cell growth. This indicates that the ability to bind and/or hydrolyze ATP is crucial for the pneumococcus and further suggests that UbK autophosphorylation could modulate ATP hydrolysis.

### The ATP Hydrolysis Activity of UbK Is Modulated by Autophosphorylation

**FIGURE 4:**
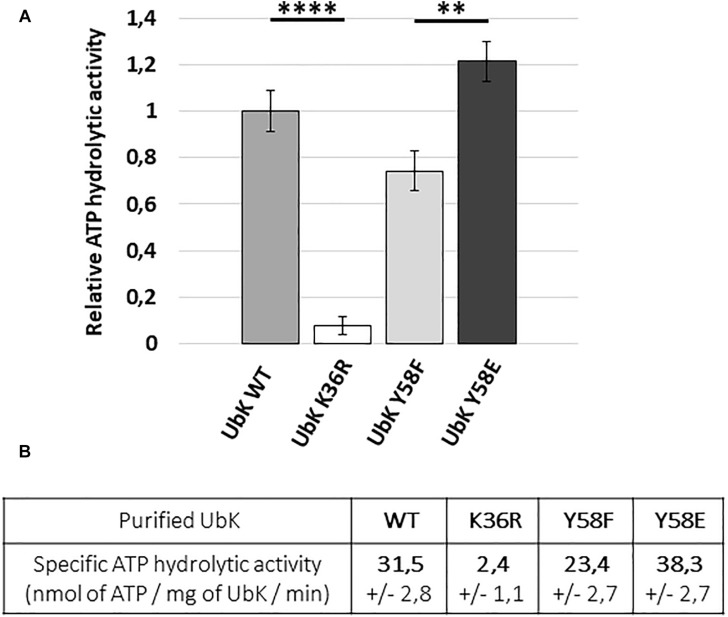
ATP hydrolytic activity of WT and mutated UbK-6His proteins. **(A)** Hydrolysis of ATP is monitored during 50 min at 37°C by measuring the disappearance of NADH at 340 nm. Bars are representative of the relative ATP hydrolytic activity. The activity of UbK WT is standardized at 1. Standard deviation is represented by vertical bars. An ANNOVA test was performed using the GraphPad software (^****^P < 0.0001 and ^∗∗^*P* < 0.005). The experiment was repeated three times. **(B)** Specific ATP hydrolytic activities of the UbK-6His proteins in nmol.mg^–1^.min^–1^

To further check our hypothesis, we analyzed the ability of UbK mutant proteins to hydrolyze ATP. For that, we overproduced in *E. coli* and purified UbK mutants fused to a C-terminal 6His-tag (UbK-6His, UbK-K36R-6His, UbK-Y58F-6His and UbK-Y58E-6His) (Supplementary Figure 4). Then, we used the pyruvate-kinase/lactate-deshydrogenase assay to measure the ATP hydrolysis activity of UbK (Karst et al., 2009; Typas et al., 2011). As expected, the inactive UbK-K36R-6His protein was unable to hydrolyze ATP (Figure 4A). By contrast, and as previously reported for Ubk of *B. subtilis*, UbK-6His harbored a low ATP hydrolysis activity (31.5 ± 2.8 nanomoles of ATP/mg of UbK/min) (Figure 4B). We further observed a significant difference regarding the ability to hydrolyze ATP between UbK-Y58F-6His and UbK-Y58E-6His. Indeed, UbK-Y58F-6His displayed a reduced activity (23.4 ± 2.7 nanomoles of ATP/mg of UbK/min) compared to UbK-6His whereas UbK-Y58E-6His hydrolyzed ATP more efficiently than UbK-6His (38.3 ± 2.7 nanomoles of ATP/mg of UbK/min). UbK autophosphorylation therefore influences its ability to hydrolyze ATP. To strengthen this observation, we incubated purified UbK-6His with radiolabeled [α-^32^P]-ATP and varying concentrations of non-radioactive ATP for different times. We observed increasing radioactive phosphorylation signals for UbK as a function of the incubation time (Supplementary Figure 5). The same experiment performed in the presence of increasing concentrations of non-radioactive ATP, showed a decrease of the UbK phosphorylation signal (Supplementary Figure 5). This reflected that non-radioactive ATP competes with the incorporation of radiolabeled [α-^32^P]-ATP. In light of the immunodetection of tyrosine autophosphorylation made with UbK purified directly from *E. coli* cells (Figure 2), it shows that purified UbK-6His contained a mix of phosphorylated and non-phosphorylated forms of UbK. ATP hydrolysis activity of UbK-6His reflects therefore the activity of both phosphorylated and dephosphorylated forms. The difference between ATP hydrolysis activities of UbK-Y58F-6His and UbK-Y58E-6His confirms that UbK dephosphorylation hampers its ability to hydrolyze ATP. Consistent with the growth defect observed for Δ*ubk*, *ubk-*K36R and *ubk*-Y58E pneumococcal mutants compared to WT and *ubk*-Y58F (Figure 3), these findings show that ATP hydrolysis by UbK is modulated by its autophosphorylation and suggest that a balanced ratio between the phosphorylated form and the non-phosphorylated form is required for the pneumococcal growth.

### *ubk* Mutants With Growth Defects All Show an Abnormal Morphology

**FIGURE 5:**
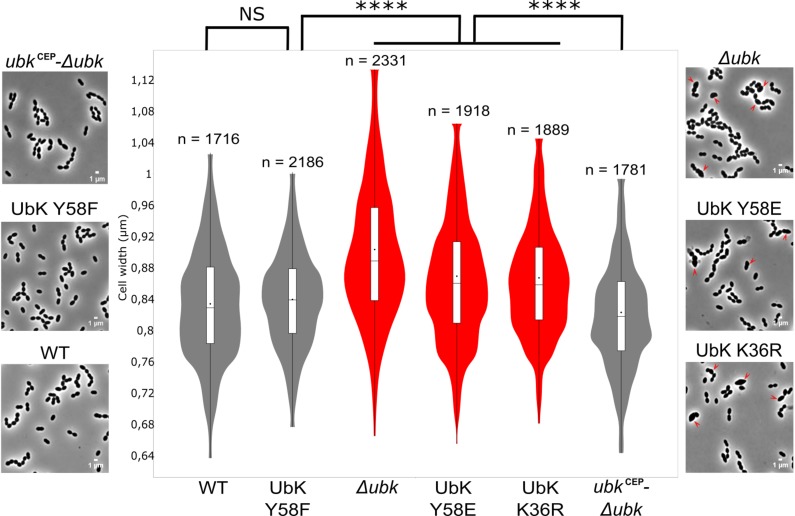
Cell morphology of *ubk* mutants and cell-width analysis. Phase contrast microscopy micrographs of the WT, Δ*ubk*, complemented (*ubk*^CEP^−Δ*ubk*), *ubk-*K36R, *ubk-*Y58F, and *ubk-*Y58E strains. Arrows show swelled mutant cells. Scale bar, 1 μm. Violin plot showing the distribution of the cell width (μm) for each strain. Mann–Whitney test (^****^P < 0.0001). The distribution of the cell width of mutants with morphological defects is shown in red. The number of cells scored and analyzed for each strain is indicated. For each violin, the width of the shaded area represents the proportion of cells located there. The bottom and top of the inside-box represent the 25th and 75th percentile. The bar in the box indicates the median value while the black dot indicates the mean value.

The growth defects of *ubk* mutants prompted us to analyze the morphology of cells by contrast phase microscopy. As shown in Figure 5, all mutants with growth defects (Δ*ubk*, *ubk-*K36R and *ubk-*Y58E) showed a phenotype characterized by cells with an aberrant morphology. Indeed, cells appeared swelled and bigger. This visual impression was confirmed statistically by analyzing a large number of cells (Figure 5). More precisely, the analysis of cell size-parameters showed that these cells are wider than for the WT cells. Consistent with the normal growth measured for *ubk-*Y58F and *ubk* complemented (*ubk*^CEP^-Δ*ubk*) cells, these cells did not display any defects and are similar to WT cells. This indicates that the slow and delayed growth of UbK mutants is likely due to a defect in cell morphogenesis and that UbK is required in this process.

**FIGURE 6:**
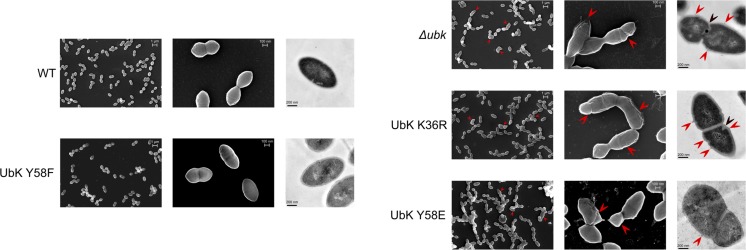
Scanning and transmission electron micrographs of WT strain and *ubk* mutants. From left to right column: scanning electron micrographs with the lowest power magnifier (scale bar, 1 μm), scanning electron micrographs with the highest power magnifier (scale bar, 100 nm) and transmission electron micrographs (scale bar, 200 nm). Red arrows on scanning electron micrographs show gashes or possible zones of peeling on the cell-wall of the mutants. Red arrows on transmission electron micrographs show zones of peeling on the cell-wall; black arrows show mispositioned septa.

The bacterial cell wall, whose main component is the peptidoglycan, is responsible of the bacterial cell shape (Typas et al., 2011). We therefore observed the cells by transmission and scanning electron microscopy to evaluate the presence of potential defects in the cell wall ultrastructure of *ubk* mutants. As shown in Figure 6, transmission electron microscopy (TEM) images showed that the integrity of the cellular envelope was compromised. Indeed, we detected small cell wall extrusion at the surface of cells, reminiscent of cell wall peeling, in Δ*ubk*, *ubk-*K36R and *ubk-*Y58E mutants. In addition, these defects were associated with an abnormal positioning and/or orientation of the septum plane and shape (Figure 6). Such defects were not observed in WT and *ubk-*Y58F cells. On the other hand, scanning electronic microscopy (SEM) further revealed the presence of gashes in the cell walls of Δ*ubk*, *ubk-*K36R and *ubk-*Y58E mutants with cells even not properly covered by the cell wall envelop at the poles (Figure 6).

**FIGURE 7:**
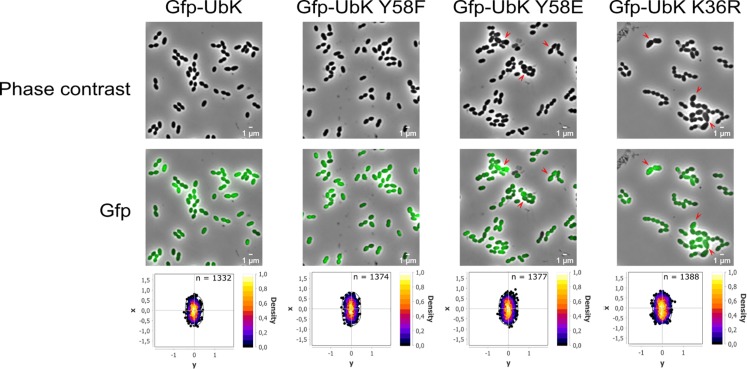
Cytoplasmic localization of the different GFP-UbK forms. Phase-contrast images **(top row)** and GFP images **(bottom row)** of exponentially growing cells expressing GFP-UbK or GFP-UbK-K36R or GFP-UbK-Y58F or GFP-UbK-Y58E. Bar scale 1 μm. Heat maps represent the localization patterns of GFP-UbK or GFP-UbK-K36R or GFP-UbK-Y58F or GFP-UbK-Y58E. The *n* values represent the number of cells analyzed.

Considering the aberrant cell shape of Δ*ubk*, *ubk-*K36R and *ubk-*Y58E mutants, we questioned the localization of UbK. We therefore analyzed the cellular localization of the diverse UbK mutant forms using fluorescence microscopy. For that, we generated strains expressing the different forms of GFP-UbK (mutated on the catalytic lysine and Y58) from the *ubk* chromosomal locus. Expression of the GFP-UbK fusions in *gfp*-*ubk*, *gfp*-*ubk* K36R, *gfp*-*ubk* Y58F and *gfp*-*ubk* Y58E strains was analyzed by Western blot using an anti-GFP antibody and showed that fusions are produced at comparable levels (Supplementary Figure 6A). In addition, these mutant strains grew and presented a similar morphology as their non-fused *gfp* counterparts (Supplementary Figures 6B,C and Figure 3). We observed that UbK localized in the cytoplasm and that phosphorylation or dephosphorylation of UbK had no impact on its localization (Figure 7). Indeed, GFP-UbK, GFP-UbK K36R, GFP-UbK Y58F and GFP-UbK Y58E all localize in the cytoplasm. Altogether, these observations indicate that the assembly of the cell wall is compromised in absence of active UbK suggesting a role in cell morphogenesis that does not rely on particular localization of UbK.

## Discussion

In this work, we have shown that UbK of *S. pneumoniae*, which belongs to a newly identified class of ubiquitous bacterial protein kinases (Nguyen et al., 2017), is crucial for the pneumococcal growth and its morphology. Opposing observations were previously reported concerning the essentiality of UbK (Freiberg et al., 2001; Kobayashi et al., 2003; Zalacain et al., 2003; Havarstein et al., 2006; Hunt et al., 2006; French et al., 2008; Karst et al., 2009; Molzen et al., 2011). We have now clarified the question for *S. pneumoniae* by showing that *ubk* deletion is actually co-selected with a single mutation (Figure 1) leading to the substitution of aspartic acid 151 for an alanine in the AsnS protein. The latter is described as a potential Asn-tRNA synthetase. AsnS enzymes are known to catalyze specific aminoacylation of tRNA_Asn_ with asparagine (Asn-tRNA_Asn_) (Hausmann and Ibba, 2008). Another indirect route to Asn-tRNA_Asn_ formation is often found in bacteria and involves the GatA/B/C subunit-proteins that transamidate Asp-tRNA_Asn_ (Akochy et al., 2004; Mladenova et al., 2014). In the R6 *S. pneumoniae* strain, both the *gatA*/*B*/*C* and the *asnS* genes are found in the genome, suggesting that *asnS* could be dispensable in order to obtain Asn-tRNA_Asn_. Nevertheless, we confirmed the essentiality of *asnS* but not that of the aspartate 151 of AsnS that can be substituted for an alanine and be suppressive for the deletion of *ubk* (Figure 1). A direct relationship between the incorporation of Asn in proteins and the morphogenesis of *S. pneumoniae* has not been reported yet. Studying the potential connection between AsnS and UbK represents therefore a promising venue to better understand the physiology of the pneumococcus in the future. It is, however, tempting to make some assumptions. Indeed, the function of AsnS might be altered or enhanced when aspartate 151 is substituted for an alanine and sufficient to render UbK dispensable. Interestingly, substitution of an aspartic acid for an alanine could mimic a phospho-ablative mutation. A recent comparative genomic study indicates that nature uses this trick by evolving serine, threonine, and tyrosine phosphorylation sites from Asp/Glu residues (Pearlman et al., 2011). An interesting speculation could be that AsnS activity could have been regulated by UbK and that this regulatory switch would have been lost in the course of evolution by the substitution of an Alanine for an Aspartic acid.

Despite *ubk* is largely but exclusively conserved in the bacterial kingdom, it is also intriguing that its genetic environment in *S. pneumoniae* is so different from that of *B. subtilis* and other bacteria like *E. coli* in which the genetic environment of *yjeE*, the *ubk* homolog, is still different (Mangat and Brown, 2008). This suggests that the cellular function of UbK could operate differently and even differ from one bacterium to another. In *S. pneumoniae*, *ubk* is described as the second gene of an operon of 4 genes, the first being *comM*. *comM* codes for a membrane immunity factor that protects competent cells against their own lytic enzymes (Havarstein et al., 2006). ComM was also shown to delay pneumococcal division during competence (Berge et al., 2017). The third *orf*, *spr1760*, codes for a protein of unknown function while the fourth codes for the essential LytR protein, a regulator of the LytR-Cps2A-Psr (LCP) protein family. LytR is proposed to belong to a type of enzymes required for the attachment of anionic cell wall polymers, like teichoic acids and capsular polysaccharides, to peptidoglycan (Kawai et al., 2011) and is also proposed to be involved in cell division and morphogenesis of the pneumococcus (Johnsborg and Havarstein, 2009). As described by Johnsborg and Havarstein (2009; Johnsborg and Havarstein, 2009), a Δ*lytR* mutant was obtained with a low frequency close to that of the Δ*ubk* mutant, confirming that *lytR* is also essential in absence of suppressive mutation(s). We constructed a Δ*lytR* strain and observed that the deletion of *lytR* did not select the same suppressive mutation as that found with *ubk* deletion (i.e., in the *asnS* gene) (data not shown). Supported by the ectopic complementation of Δ*ubk*, this substantiates distinct roles of UbK and LytR in morphogenesis and/or morphology.

Based on the absence of *ubk* only in few *Mycoplasma* or *Ureaplasma*, that are devoid of cell-wall, and because the *ubk* homolog *yjeE* is potentially co-transcribed with an amidase gene in *H. influenzae*, it had been proposed that UbK would be implicated in the cell-wall assembly (Teplyakov et al., 2002). Nevertheless, if these arguments constitute clues, they are not direct proofs to assign a role to UbK in cell-wall homeostasis. Our findings that mutations or deletion of *ubk* affect the morphology of *S. pneumoniae* (Figures 5, 6) however support this claim and strongly suggest that UbK is required for the cell wall assembly in the pneumococcus.

An important feature is also that Y58 of UbK in not conserved in YdiB of *B. subtilis* (Nguyen et al., 2017). Indeed, the tyrosine 58 is replaced by a phenylalanine (F63). Considering that YdiB autophosphorylates mainly on Ser and Thr (Nguyen et al., 2017), and in view of all the observations discussed above, one can suggest that the mode of regulation of UbKs as well as their cellular functions have evolved independently to operate differently and to potentially serve distinct purposes. To strengthen this point of view, we tried to functionally complement the Δ*ubk* pneumococcal mutant with the WT or F63Y alleles of the *ydiB* gene of *B. subtilis* but none of the two alleles allowed complementing the growth defects due to *ubk* deletion in *S. pneumoniae* (data not shown).

**FIGURE 8:**
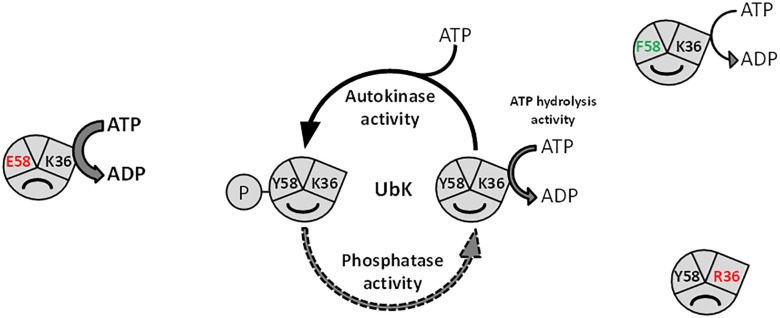
Model depicting the function of UbK as an ATP hydrolytic enzyme and as a tyrosine-autokinase. For each version of UbK, the ATP hydrolysis activity is shown with an arrow whose size depends on the relative level of this enzymatic activity. WT UbK, mutants of the lysine 36 of the Walker A motif, as well as mutants of the phospho-site (Tyrosine 58) are represented with a “smile” illustrating whether the mutation is beneficial or detrimental for the cell growth and the morphology of *S. pneumoniae*.

We have also shown that the ability of UbK to hydrolyze ATP prevails over its autophosphorylation since a phosphoablative mutant (*ubk-*Y58F) grows like a WT strain and does not display the morphological defects of the catalytic *ubk-*K36R mutant or the *ubk-*Y58E mutant. In addition, the K36R mutation is epistatic toward the Y58F or Y58E mutations since double mutants grow like the *ubk-*K36R mutant. This allows us to propose a model in which the level of initial phosphorylation enhances and determines the level of ATP hydrolysis activity and subsequently the ability of the cells to grow properly. This model also implies that only a moderate range of ATP hydrolysis by UbK is required by the cell (Figure 8). In this model, one can speculate that a phosphatase, not yet identified, could consistently adjust the level of UbK phosphorylation and consequently the enhancement of UbK ATP hydrolysis. To date, the only well-known phosphatase of *S. pneumoniae* susceptible to play such a role is CpsB, the cognate phosphotyrosine-phosphatase of the BY-kinase CpsD (Standish et al., 2013). Nevertheless, as for protein-kinases, one cannot exclude that other types of atypical phosphatase idiosyncratic to bacteria may exist and that a specific phosphatase works together with UbK. Similarly, one cannot exclude the existence of potential phosphorylation substrate(s) of UbK that still remain unknown. However, we never obtained either *in vivo* evidence of UbK-mediated phopsphorylation of an endogenous substrate (by probing whole cell lysates with anti-phosphotyrosine antibodies, Supplementary Figure 1B), nor *in vitro* UbK-mediated phosphorylation signals for proteins found to be phosphorylated on Tyr in the phosphoproteome (Sun et al., 2010) (data not shown). Altogether, these data suggest that UbK of *S. pneumoniae* could either act as a substrateless-kinase whose autophosphorylation level is crucial for the interaction with its partners and/or as a kinase phosphorylating with low efficiency some endogenous substrates. Whatever the case, UbK autophosphorylation and/or phosphorylation of its potential targets should be finely controlled for normal growth and morphogenesis of *S. pneumoniae*. Further phosphoproteomic studies are needed to gain a better understanding on the level of occupancy of the phosphosite of UbK and its potential substrates.

Until now, *S. pneumoniae* was thought to possess only one Y-kinase (CpsD) and one S/T-kinase (StkP). Our characterization of UbK supports the concept that bacteria could have evolved other types of protein-kinases that will be first identifiable only biochemically. In the long term, it would therefore come as no surprise if all the phosphoproteins identified by high-throughput phosphoproteomic studies (84 in the phosphoproteome of *S. pneumoniae*, Sun et al., 2010) are phosphorylated by different types of protein-kinases, potentially forming a complex and interconnected regulatory network indispensable for the biology of the pneumococcal cell.

## Materials and Methods

### Bacterial Strains, Plasmids and Oligonucleotides

Strains and plasmids, and primers used in this study are listed in Supplementary Material as Supplementary Tables 1, 2 respectively.

### Growth Conditions, Media and Bacterial Transformation

*Streptococcus pneumoniae* strains were cultivated at 37°C in Todd-Hewitt Yeast (THY) broth (Difco) or in C + Y medium (Martin et al., 1995). For growth curves, cells were previously grown in THY to an OD of 0.5 at 550 nm and aliquoted. From a frozen aliquot, cells were count and numbered by plating onto THY-agar plates. Cells were then diluted so that 2.10^5^ to 10^6^ bacteria/mL were inoculated in a fresh THY or C + Y liquid medium at 37°C. *S*. *pneumoniae* mutants were constructed by transformation of the WT strain or derivatives as previously described (Martin et al., 2000) using precompetent cells treated at 37°C during 30 min with the synthetic competence stimulating peptide 1 (CSP1) at the concentration of 100 ng/10^8^ cells to induce competence. Cells were then plated on THY-agar supplemented with 3% (vol/vol) defibrinated horse blood and incubated for 120 min at 37°C. Selection of transformants was then performed by adding a THY-agar overlay containing the appropriate antibiotic (streptomycin 200 μg.mL^–1^, kanamycin 250 μg.mL^–1^, spectinomycin 100 μg.mL^–1^) and further incubated for either 16 or 30 h at 37°C. The strains expressing an ectopic copy of *ubk*, *gfp*-*ubk*, *gfp*-*ubk*K36R or *gfp*-*ubk*Y58F at the *amiF/treR* locus under the control of the maltose inducible promoter P_M_ (Guiral et al., 2006) were grown in THY or in C + Y medium containing either glucose or 1% maltose.

When transformation assays were conducted to compare the ability of different strains to transform different DNAs carrying *ubk* deletion, CSP was used to induce competence at a saturating concentration of 100 ng/10^8^ cells and a genomic control DNA (either DNA from the Δ*phpP-stkP*:*spc*-*rpsL*, or the Δ*spr1424*:*kan*-*rpsL* mutant) was used as a reference in order to standardize the transforming ability of the recipient strains.

The *E*. *coli* XL1-Blue strain was used as a host for cloning. *E*. *coli* BL21 (DE3) strain was used as a host for overexpression. Luria-Bertani (LB) broth and agar supplemented with appropriate antibiotic (tetracycline 15 μg.mL^–1^, ampicillin 100 μg.mL^–1^) were used for routine growth at 37°C. Strains used in this study are listed in Supplementary Table 1.

### Allelic Replacement Mutagenesis

To construct pneumococcus mutants (gene deletions, *gfp*-*ubk* fusions or site-directed mutagenesis), we used a two-step procedure based on a bicistronic *kan-rpsL* cassette called Janus (Sung et al., 2001) or a *spc*-*rpsL* cassette that we constructed on purpose called Janus2. The *spc*-*rpsL* Janus2 cassette was constructed by amplification of the *aad9* gene from the pR412 plasmid (a gift from J.P. Claverys’ laboratory) and fused to the *rpsL* gene. A strain containing the *kan-rpsL* cassette in its genome was then transformed with the PCR fusion fragment that allowed the substitution of the *kan-rpsL* cassette with the *spc*-*rpsL* cassette. The *spc*-*rpsL* cassette was then PCR amplified from the genomic DNA of this strain with the same primers as for the *kan-rpsL* cassette and used in the two-step Janus technique. Throughout this study, gene mutagenesis (*ubk* or *spr1397*) or fusions with *gfp* (*ubk*) were constructed at each native chromosomal locus. The mutated or fusion genes were thus expressed under the control of the native promoter and represented the only source of protein. Description of primers used for the construction of strains is provided in Supplementary Table 2. The full procedure is described in Fleurie et al. (2012). Briefly, the Janus or Janus2 cassette is used to replace the gene of interest. After transformation and selection, this confers resistance to kanamycin or spectinomycin and dominant streptomycin sensitivity (Kan^R^–Str^S^ or Spc^R^–Str^S^) in an initial WT *rpsL1* genetic background. Then, a DNA fragment flanked on each end by sequences homologous to the upstream and downstream regions of the gene of interest is used to transform and substitute the Kan^R^–Str^S^ or Spc^R^–Str^S^ in the Kan^R^ or Spc^R^ strains. Integration of the fragment is selected in final non-polar markerless mutant strains by streptomycin resistance.

The gene encoding the monomeric GFP was from Martin et al. (2010). For allelic replacement of *spr1397*, which is an essential gene, by *spr1397* D121A, we used a one-step procedure without any selection and direct enzymatic digestion of a PCR fragment amplified from genomic DNA of each selected clone after transformation. Briefly, the introduction of the D121A mutation was concomitant with the introduction of an *Alu*I restriction site in the recombinant PCR fragment covering upstream to downstream regions of *spr1397*. Characterization of the *Alu*I restriction profile of the PCR fragments covering the *spr1397* region of transformants led to identify the *Alu*I restriction site. The presence of the suppressive D121A mutation in these transformants was further confirmed by direct DNA sequencing of the *spr1397* locus.

### Complementation and/or Introduction of an Ectopic Copy of *ubk*

To obtain ectopic copies of *ubk*, *gfp*-*ubk*, *gfp*-*ubk* K36R or *gfp*-*ubk* Y58F at the *amiF/treR* locus under the control of the maltose inducible promoter P_M_ (Guiral et al., 2006), we cloned an amplified copy of *ubk* in the pCEP plasmid that was transformed directly and integrated in the WT strain to generate the *ubk*^+^-*ubk*^CEP^ strain (Guiral et al., 2006). We did the same with the *gfp* gene that was integrated into the WT reference strain to generate the *gfp*^CEP^ strain. From these two strains PCR fusion fragments using primers designed to introduce single mutations were generated. These PCR fragments (*gfp*-*ubk*, *gfp*-*ubk*K36R or *gfp*-*ubk*Y58F) contained the upstream and downstream *amiF* and *kan*-*treR* regions of the site of integration of the CEP platform respectively. Transformation of the Δ*ubk* mutant with these PCR fragments allowed obtaining the Δ*ubk*- *gfp*-*ubk*^CEP^, Δ*ubk*- *gfp*-*ubk*K36R^CEP^ and Δ*ubk*- *gfp*-*ubk*Y58F^CEP^ strains from which the GFP-UbK fusion proteins were purified. When necessary, the absence of any rearranged copy of *ubk* in the genome of transformants was controlled by PCR with the 13/14 primer pair internal to *ubk* (Supplementary Table 2).

### Construction of Plasmids

DNA fragments coding for UbK were obtained by PCR using the chromosomal DNA of our reference WT strain as template. To introduce the different mutations corresponding to UbK K36R, UbK Y58F and UbK Y58E, we used each time a specific primer overlapping the mutated region. When introducing a single mutation, chimera DNA fragments using primers introducing the mutation were amplified. Then, these chimera DNA fragments were used as a primer for a second round of PCR to amplify the whole sequence. The fragments were then cloned between the *Nde*I and *Pst*I, BamH1 and *Hin*dIII, and *Nde*I and *Bam*HI restriction sites of the pT7.7 (Cortay et al., 1994), pQE30 and pETphos plasmids respectively. The nucleotide sequences of all DNA fragments were sequenced to ensure error-free amplification. Plasmids and primers used in this study are listed in Supplementary Tables 1, 2 respectively.

### Whole Genome Sequencing

For each bacterial strain (WT and two independent *ubk* mutants), extracted genomic DNA was sheared (1 μg) following the manufacturer instructions using the S220 focused ultrasonicator (Covaris) to obtain a fragment distribution length from 100 bp to 1 kb, with an average peak around 400 bp. Each fragmented DNA material was then used to build a barcoded library using the Ion Xpress Plus gDNA (genomic DNA) Fragment Library Preparation kit (Thermofisher) following the protocol of the kit. Libraries were then size-selected using the E-gel Electrophoresis system (Invitrogen) in order to select fragments from 350 to 450 bp in length. Libraries were qualified according to the concentration and distribution profile using the TapeStation 2200 (Agilent). Diluted libraries (26 pM) were mixed at an equimolar range and were amplified through emulsion PCR using the Ion PGM Template OT2 400 kit (Thermofisher). Finally, the enriched libraries were loaded into a 316v2 chip and sequenced on the Ion PGM sequencer with the Ion PGM HiQ chemistry.

### Protein Purification

Recombinant plasmids overproducing the UbK, UbK-K36R, UbK-Y58F, and UbK-Y58E proteins were transformed into BL21 (DE3) *E*. *coli* strain. The transformants were grown at 37°C until the culture reached an OD_600_ = 0.5. Expression was induced with IPTG to a final concentration of 0.5 mM and incubation was continued for 3 h. After 3 h culture at 37°C, cells were harvested and resuspended in buffer A (Tris-HCl 50 mM, pH 7.5; NaCl 300 mM; DTT 1 mM; imidazole 10 mM; glycerol 10%) containing 10 mg.L^–1^ of lysozyme and 6 mg.L^–1^ of DNase I and RNase A, and sonicated. After centrifugation at 15000 *g* for 30 min, the supernatant was loaded onto a Ni-NTA agarose column (Qiagen) and extensively washed with buffer A supplemented with 30 mM imidazole. Samples were eluted with buffer A supplemented with 300 mM imidazole. The fractions corresponding to the pure protein were pooled and dialyzed against the following buffer: HEPES 50 mM, pH 7.5; NaCl 100 mM; DTT 1 mM; MgCl_2_ 1 mM; glycerol 10%. The protein concentrations were determined using a Coomassie Assay Protein Dosage Reagent (Uptima) and aliquots were stored at −80°C.

### GFP-Trap Method

The Δ*ubk gfp*-*ubk*^CEP^, Δ*ubk gfp*-*ubk* Y58F*^CEP^*, and Δ*ubk gfp*-*ubk* K36R*^CEP^* strains were grown at 37°C in THY medium until OD_550_ = 0.5 in presence of 1% maltose. After centrifugation at 5000 *g* for 10 min at 4°C, cell pellets were resuspended in 0,1 M Tris-HCl, 2 mM MgCl_2_, 1 M sucrose, 0,1% Triton X100. After 30 min at 30°C and centrifugation at 13000 *g* for 20 min at 4°C, cells were incubated in a hypotonic buffer (0.1 M Tris-HCl, 1 mM EDTA, 0.1% Triton X100) during 15 min at 25°C. The lysate was centrifuged at 5000 *g* for 5 min at 4°C and incubated for 2 h at 4°C with the GFP-trap slurry as recommended by the GFP-Trap^®^ A Chromotek protocol. After a centrifugation at 2700 *g* for 2 min at 4°C, the pellet was washed in a 10 mM Tris-HCl, 150 mM NaCl, 0.5 mM EDTA 0.1% Triton X100 buffer and eluted directly in 2X Laëmmli loading buffer before loading onto a SDS polyacrylamide gel electrophoresis.

### Preparation of *S. pneumoniae* Protein Lysates

Cultures of *S. pneumoniae* cells were grown at 37°C in THY medium until OD_550_ = 0.3. After centrifugation at 5000 *g* for 10 min at 4°C, cell pellets were resuspended in 25 mM Tris-HCl, 1 mM EDTA. Cells were disrupted by sonication. The protein concentration was determined by the Bradford method using the Pierce protein assay reagent.

### Immunoblot Analysis

*In vivo* UbK autophosphorylation of GFP-UbK trapped proteins of *S*. *pneumoniae* was immunodetected using a mouse anti-phosphotyrosine monoclonal antibody 4G10 (Sigma-Aldrich) at 1/2000. Detection of GFP fusions was performed using a rabbit anti-GFP polyclonal antibody (AMS Biotechnology) at 1/10000. An anti-mouse secondary polyclonal antibody horseradish peroxidase (HRP) conjugated (Biorad) was used at 1/5000 to reveal immunoblots. Detection of enolase was performed using a rabbit anti-enolase polyclonal antibody at 1/250000.

### Autophosphorylation Assays With Radiolabeled ATP

*In vitro* phosphorylation of 2 μg of UbK-6His, 6His-UbK or 6His-UbK K36R was carried out for 2–20 min at 37°C in the presence of 0 to 1 mM ATP with 125 μCi/ml [α-^32^P]ATP as previously described (Vincent et al., 1999). After electrophoresis, radioactive proteins were visualized by autoradiography.

### ATP Hydrolysis Activity

The enzymatic assay used to measure the ATP hydrolysis activity couples the regeneration of ATP from the ADP produced to the conversion of phosphoenolpyruvate (PEP) to pyruvate by pyruvate kinase (PK) and the conversion of pyruvate to lactate by lactate dehydrogenase (LDH) (Jault et al., 1991). One mole of ATP hydrolyzed is directly converted to one mole of NADH oxidized to NAD^+^, and the ATP hydrolysis activity is monitored by the disappearance of NADH, followed at 340 nm. Specific activities are calculated from the Beer-Lambert law using the molar extinction coefficient of NADH (6200 mol.L^–1^.cm^–1^) and expressed in nmol of ATP.mg^–1^ of UbK.min^–1^. Mixtures were prepared as described by Karst et al. (2009).

### Nano LC-MS/MS Analysis of Purified UbK

Purified UbK was in-gel digested using trypsin as described in Borchert et al. (2010) and peptide mixture was desalted using C18 StageTips (Rappsilber et al., 2007). Analysis of peptides was done on a Proxeon Easy-LC system (Proxeon Biosystems) coupled to an LTQ-Orbitrap-Elite mass spectrometer (Thermo Fisher Scientific) equipped with a nanoelectrospray ion source (Proxeon Biosystems) as described previously (Conzelmann et al., 2013).

Mass spectra were analyzed using the software suite MaxQuant, version 1.3.0.5 (Cox and Mann, 2008). Extracted peak lists were submitted to database search using the Andromeda search engine (Cox et al., 2011) to query target-decoy (Elias and Gygi, 2010) databases consisting of 35,214 protein entries from *S. pneumoniae* serotype 2 strain D39/NCTC 7466, and 2,029 protein entries from *S. pneumoniae* strain ATCC BAA-255, both obtained from Uniprot and against the sequence of the His-tagged protein UbK and 248 commonly observed contaminants. Trypsin was defined as protease and two missed cleavage sites were allowed. Acetylation at the N-terminus, oxidation of methionine and phosphorylation on serine, threonine, and tyrosine were set as variable modifications. Carbamidomethylation of cysteine was set as fixed modification. Initial precursor mass tolerance was set to six parts per million (ppm) at the precursor ion level and 20 ppm at the fragment ion level. Spectra of modified peptides were manually validated.

### Microscopy Techniques

GFP fusion of wild-type UbK or mutants were visualized by fluorescence microscopy using *S. pneumoniae* cell culture at OD_550 nm_ = 0.1. Slides were visualized with a Nikon TiE microscope fitted with an Orca-CMOS Flash4 V2 camera with a 100 × 1.45 objective. Images were collected using NIS-Elements (Nikon). Images were analyzed using the software ImageJ^1^ and the plugin MicrobeJ (Ducret et al., 2016).

For TEM, *S. pneumoniae* cells were grown at 37°C to an OD of 0.1 in THY medium. Samples were then collected, centrifuged and fixed overnight with 5% glutaraldehyde in 0.1 M cacodylate buffer (pH 7.5) at 4°C. Postfixation with 1% osmium tetroxide in cacodylate buffer was carried out for 1 h at room temperature. These fixed cells were dehydrated using a graded series of ethanol and embedded in LR-white at 60°C for 48 h. Ultrathin sections (60 nm) were obtained using a Leica UC7 microtome and were counterstained with uranyl acetate and lead citrate (Reichert Ultrostainer, Leica, Germany). Samples were examined with a Philips CM120 transmission electron microscope quipped with a Gatan Orius SC200 CCD camera.

For SEM, cells were grown at 37°C to an OD of 0.1 in THY medium, spotted onto poly-lysine coverslips, washed with phosphate-buffered saline, and fixed in 0.18 M cacodylate buffer (pH 7.6) containing 2% glutaraldehyde. All samples were then dehydrated with a graded series of ethanol, passage in HMDS (1,1,1,3,3,3-hexamethyldisilazane) and finally air dried. The dried samples were covered with a 10 nm-thick gold/platinum layer (MED20, Baltec). Samples were then observed with a Quanta 250 FEG (FEI) scanning electron microscope.

## Data Availability

The genomic data generated can be found in the NCBI Sequence Read Archive (PRJNA562556).

## Author Contributions

AP, SG, CF, CGa, and JT conducted all the experiments of cell biology, genetics, purified proteins, and performed the ATP hydrolysis assays and western blot analysis. CC performed the transmission electron microscopy images. MF-W and BM performed the mass spectrometry experiments. AP, SG, CF, CGa, and CC designed and analyzed the data together with SG, J-MJ, and CGr. SG and CGr wrote the manuscript. All authors edited the manuscript.

## Conflict of Interest Statement

The authors declare that the research was conducted in the absence of any commercial or financial relationships that could be construed as a potential conflict of interest.

## Footnotes

1 http://rsb.info.nih.gov/ij/
