# Supplementary material for: The Tyrosine-Autokinase UbK Is Required for Proper Cell Growth and Cell Morphology of Streptococcus pneumoniae
Source: Front Microbiol. 2019 Sep 3;10:1942. doi: 10.3389/fmicb.2019.01942 (PMC6733980; doi:10.3389/fmicb.2019.01942)
Supplement: Supplementary file 1 [file Data_Sheet_1.PDF]

Supplementary material

Supplementary tables

Supplementary table 1. Strains and plasmids used in this study

Strains	Genotype and description	References
S. pneumoniae strains		
R800	*S. pneumoniae* R6 derivative strain	Gift from J.-P. Claverys, Toulouse, France
WT	R800 *rpsL1* ; Str^{R}	(Fleurie et al., 2012)
Δ*ubk*::*kan-rpsL*	Δ*ubk*::*kan-rpsL*, *spr1397*D151A ; Kan^{R} ; original *ubk* mutant with *ubk* suppressive mutation	This study
Δ*ubk*::*spc-rpsL*	Δ*ubk*::*spc-rpsL*, *spr1397*D151A ; Spc^{R} ; original *ubk* mutant with *ubk* suppressive mutation	This study
Δ*ubk*	Δ*ubk*, *spr1397*D151A ; Str^{R}	This study
ubk^{+}-*ubk*^{CEP}	CEP[P_m *ubk*] ; Kan^{R}, Str^{R}	This study
Δ*ubk*::*spc-rpsL* -*ubk*^{CEP}	Δ*ubk*::*spc-rpsL*, CEP[P_m *ubk*] ; Kan^{R}, Spc^{R}	This study
Δ*ubk*-*ubk*^{CEP}	Δ*ubk*, CEP[P_m *ubk*] ; Kan^{R}, Str^{R}	This study
suppressor-WT	*spr1397*D151A ; Str^{R}	This study
gfp^{CEP}	CEP[P_m *gfp*] ; Kan^{R}, Str^{R}	This study
Δ*ubk gfp-ubk*^{CEP}	Δ*ubk*, CEP[P_m *gfp-ubk*] , spr*1397*D151A ; Kan^{R}, Str^{R}	This study
Δ*ubk gfp-ubk*K36R^{CEP}	Δ*ubk*, CEP[P_m *gfp-ubk*K36R] , spr*1397*D151A ; Kan^{R}, Str^{R}	This study
Δ*ubk gfp-ubk*Y58F^{CEP}	Δ*ubk*, CEP[P_m *gfp-ubk*Y58F] , spr*1397*D151A ; Kan^{R}, Str^{R}	This study
*ubk*K36R	*ubk*K36R, *spr1397*D151A ; Str^{R}	This study
*ubk*Y58F	*ubk*Y58F, *spr1397*D151A ; Str^{R}	This study
*ubk*Y58E	*ubk*Y58E, *spr1397*D151A ; Str^{R}	This study
gfp-ubk	*gfp-ubk*, *spr1397*D151A ; Str^{R}	This study
*gfp-ubk*K36R	*gfp-ubk*K36R, *spr1397*D151A ; Str^{R}	This study
*gfp-ubk*Y58F	*gfp-ubk*Y58F, *spr1397*D151A ; Str^{R}	This study
*gfp-ubk*Y58E	*gfp-ubk*Y58E, *spr1397*D151A ; Str^{R}	This study
*ubk*K36R-Y58F	*ubk*K36R-Y58F, *spr1397*D151A ; Str^{R}	This study
*ubk*K36R-Y58E	*ubk*K36R-Y58E, *spr1397*D151A ; Str^{R}	This study
Δ*phpP-stkP*::*spc-rpsL*	Δ*phpP-stkP*::*spc-rpsL* ; Spc^{R}	This study
Δ*spr1424*::*kan-rpsL*	Δ*spr1424*::*kan-rpsL* ; kan^{R}	This study
E. coli strains		
XL1-Blue	*sup*E44 *hsd*R17 *rec*A1 *end*A1 *gyr*A46 *thi rel*A1 lac- F′[*pro*AB+ *lac*Iq *lac*ZΔM15 Tn10 (Tc^{R})]; Tet^{R}	(Bullock et al., 1987)

BL21(DE3)	F$^-$ *ompT* *gal* *dcm* *lon* *hsd*SB (rB$^-$ mB$^-$) λ (DE3 [*lac*I *lac*UV5-T7 gene 1 *ind*1 *sam*7 *nin*5])	(Studier and Moffatt, 1986)
Plasmids		
pUC57-*gfp*	pUC57 derivative, encoding the *gfp* gene, from Met1 to Lys239, Amp^R	(Martin et al., 2010)
pT7.7	pT7.7 derivative, encoding a His-tag for C-terminal fusions; Amp^R	(Cortay et al., 1994)
pT7.7 *ubk*	pT7.7 derivative, encoding *ubk*; Amp^R	This study
pT7.7 *ubk*Y58F	pT7.7 derivative, encoding *ubk*Y58F; Amp^R	This study
pT7.7 *ubk*Y58E	pT7.7 derivative, encoding *ubk*Y58E; Amp^R	This study
pT7.7 *ubk*K36R	pT7.7 derivative, encoding *ubk*K36R; Amp^R	This study
pQE30 *ubk*	pQE30 derivative, encoding *ubk*; Amp^R	This study
pETphos *ubk*K36R	pETphos derivative, encoding *ubk*K36R; Amp^R	This study
pR412	Donor for Spc^R cassette, Amp^R, Spc^R	Gift from J.-P. Claverys and M. Prudhomme, Toulouse, France

Supplementary table 2. List of primers

Purpose	Gene or plasmid	N°[a] and name	Sequence 5'-3'[b], gene, position[c]
Construction of *S. pneumoniae* strains	Janus cassettes	1 Janus (+)	CCGTTTGATTTTTAATGGATAATG, upstream of *kan* or *aad9* in Janus or Janus2 cassettes, -70 or -144 respectively
		2 Janus (-)	AGAGACCTGGGCCCCTTTCC, downstream of *kan* or *aad9* in Janus or Janus2 cassettes, +1341 or +1301 respectively
	ubk or *ubk-gfp* at *ubk* genuine locus or on CEP platform	3 up *ubk* (+)	TATGGATCCAAATCAATGAGAATCTTATTTTTGTTAGC, upstream of *ubk*, -703
		4 *ubk* (-)	CATTATCCATTAAAAATCAAACGGACTCTTATTATACCAAAAACTTTTCTTTTGTG, *ubk*, 0
		5 *ubk* (+)	GGAAAGGGGCCCAGGTCTCTATGGAGTATGAATTGCTCATTAGGG, *ubk*, +433
		6 down *ubk* (-)	ATACCTCCTACTGCATCCAC, downstream of *ubk*, +1444

		7 *ubk* (-)	CCCTAATGAGCAATTCATACTCCATACTCTTATTATACCAAAAACTTTTCTTTTGTG, *ubk*, 0
		8 *ubk* (+)	ATGGAGTATGAATTGCTCATTAGGG, *ubk*, +433
		9 *gfp-ubk* (-)	CAATTCTTCACCTTTAGAAACCATACTCTTATTATACCAAAAACTTTTCTTTTG, *gfp*, 0
		10 *gfp-ubk* (+)	GTATAAA**CTCGAGGGATCCGGA**ATGTACACAAAAAATGAAGAAGAGTTGC, *ubk*, 0
		11 *gfp* (+)	ATGGTTTCTAAAGGTGAAGAATTG, *gfp*, 0
		12 *gfp* (-)	**TCCGGATCCCTCGAG**TTTATACAATTCATCCATACCATG, *gfp*, +717
		13 *ubk* (+)	TAATAAGA*CCATGG*ACACAAAAAATGAAGAAGAGTTGC, *ubk*, 0 (NcoI)
		14 *ubk* (-)	TAT*GGATCC*TCATACTCCATATTGAAGCTCC, *ubk*, +444 (BamHI)
	gfp on CEP platform	15 *gfp* (-)	ATGAATTC*CCATGG*TTTCTAAAGGTG, *gfp*, 0 (NcoI)
		16 *gfp* (+)	TAT*GGATCC*TTATTTATACAATTCATCCATACCATGTG, *gfp*, +720 (BamHI)
	spr1397	17 up *spr1397* (+)	GCAGCAGTTGAGGCTCTATCAGG, upstream of *spr1397*, -733
		18 *spr1397* (-)	CATTATCCATTAAAAATCAAACGGTATTTTTCCTTTTCTTTTTTATTCTTTATGGC, *spr1397*, 0
		19 *spr1397* (+)	GGAAAGGGGCCCAGGTCTCTGAACAGGAAAACGCCCATGTGG, *spr1397*, +1341
		20 down *spr1397* (-)	GCCTGTTGGCGCCACATAACG, downstream of *spr1397*, +1971
		21 up *spr1397* (+)	GTGAGAATGATTGATCATTTTGAGATTAAG, upstream of *spr1397*, -391
		22 *spr1397* (-)	TATCCATCAAAAA***AGCT***GTTCCGTGTTC, *spr1397*, +376 (AluI)
		23 down *spr1397* (-)	GGTAAGTGAGGTTCGCTCAC, downstream of *spr1397*, +1449
Construction of plasmids for protein expression in *E. coli*	pT7.7-*ubk*	24 *ubk* (+)	GGGAATTC*CATATG*TACACAAAAAATGAAGAAGAGTTG, *ubk*, 0 (NdeI)
		25 *ubk* (-)	TAT*CTGCAG*TACTCCATATTGAAGCTCCTCTAAC, *ubk*, +441 (PstI)
		26 *ubk* (+)	TAT*GGATCC*TACACAAAAAATGAAGAAGAGTTG, *ubk*, 0 (BamHI)
		27 *ubk* (-)	TAT*AAGCTT*TCATACTCCATATTGAAGCTC, *ubk*, +441 (HindIII)
		28 *ubk* (-)	TAT*GGATCC*TCATACTCCATATTGAAGCTCCTC, *ubk*, +441 (BamHI)
		29 *ubk*K36R (-)	AAGGTCGTT**CG**ACCTGCACC, *ubk*, +117
		30 *ubk*Y58F (+)	GTCCCACCT**T**TACTATCGTG, *ubk*, +164
		31 *ubk*Y58E (+)	GTCCCACC**G**A**A**ACTATCGTG, *ubk*, +164

[a] Forward and reverse primers are represented by plus (+) or minus (-), respectively.

[b] For primer pairs 1/4, 2/5, 7/8, 9/11, 10/12, 1/18 and 2/19, sequences underlined are complementary to each other. The sequences in bold in primers 10 and 12, inserted between the *gfp* and *ubk* genes, code for a linker between GFP and UbK. For primers 13 to 16, 22, 24 to 28 restriction sites are italicized and the corresponding restriction enzymes are indicated into brackets. For primers 22 and 29 to 31, mutated bases are in bold.

[c] - and + indicate respectively upstream and downstream positions relative to the ATG codon of the corresponding gene.

Supplementary figures

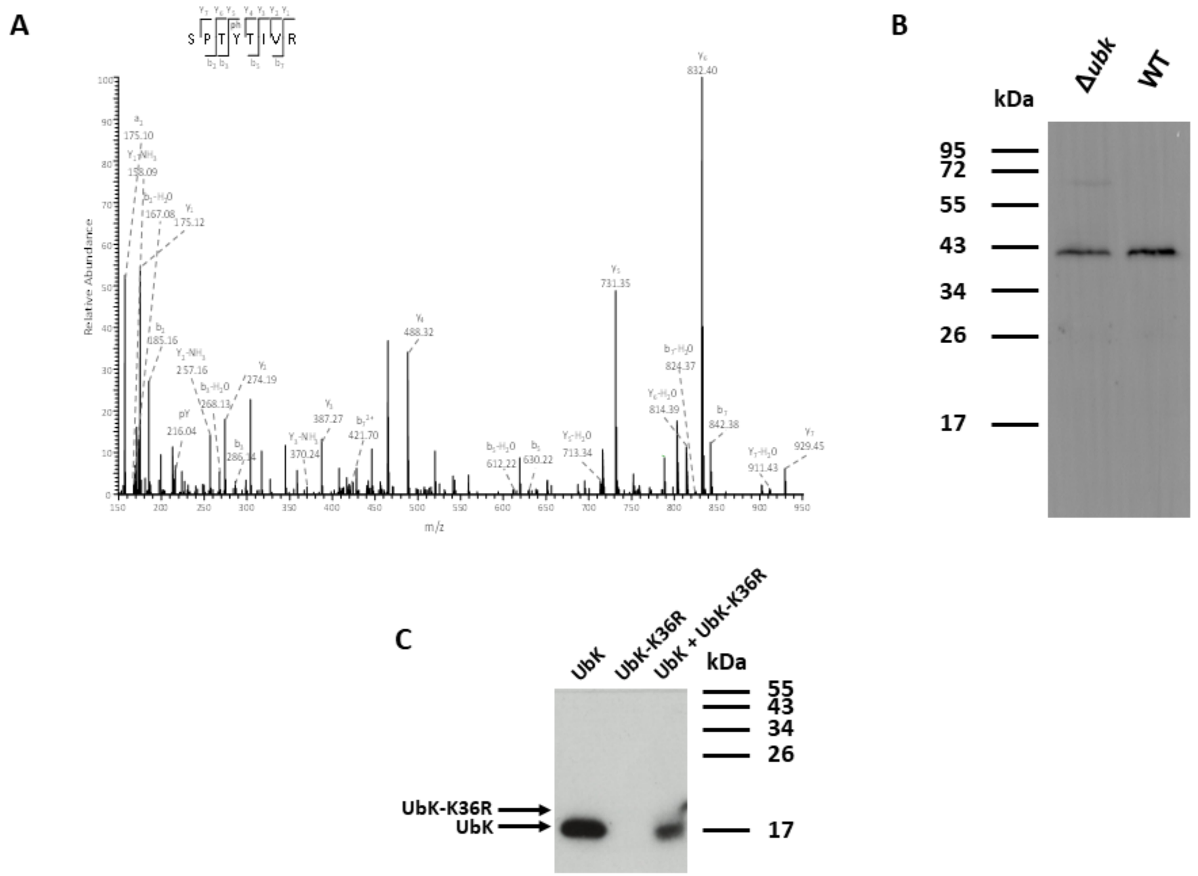

Supplementary figure 1.
LC-MS/MS phosphorylation analysis of GFP-UbK purified from *S. pneumoniae*, immunodetection of potential substrates of UbK and autoradiography of cis-autophosphorylation of UbK

A. Purified GFP-tagged UbK was digested in gel with trypsin, and peptide mixture was analysed by LC-MS/MS. UbK is phosphorylated on tyrosine 58. The spectrum shows the fragmentation pattern of the phosphopeptide SPTY(ph)TIVR corresponding to amino acids 55-62.

B. Western immunoblot of whole cell lysates from strains Δ*ubk* and WT probed with an anti-phosphotyrosine antibody.
C. UbK purified from *E. coli* with a 1.3 kDa 6His tag was incubated at 37° C during 20 min with [α-^{32}P]-ATP and UbK-K36R purified from *E. coli* with a 2.7 kDa 6His tag. Mixtures were then analyzed by SDS-PAGE and autoradiography. Arrows show the location of the UbK and UbK-K36R bands revealed with Coomassie blue staining.

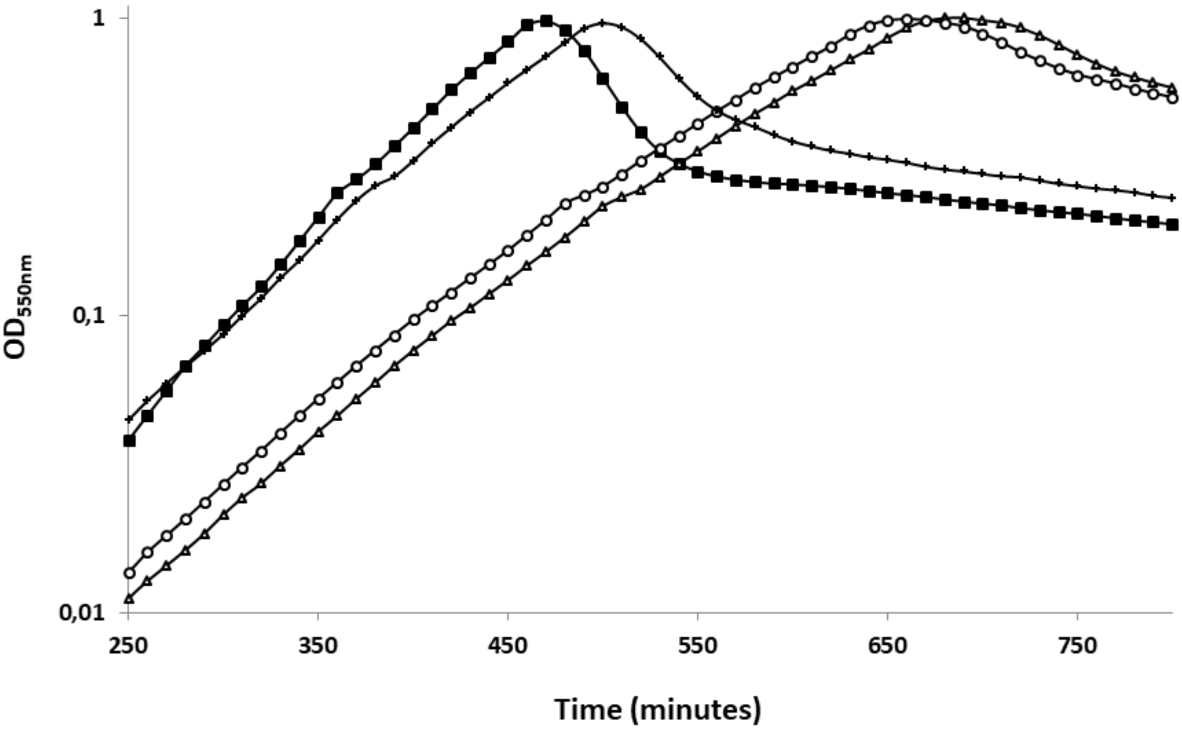

Supplementary figure 2.
Representative growth curve of the *suppressor*-WT strain
Comparative growth curves at 37°C of the WT (black squares), Δ*ubk* (white triangles), *ubk*-K36R (white circles), *suppressor*-WT (black crosses) strains. Bacteria were diluted so that 2.10^5 cells were inoculated at t=0 min.
Growths were led in triplicate.
.

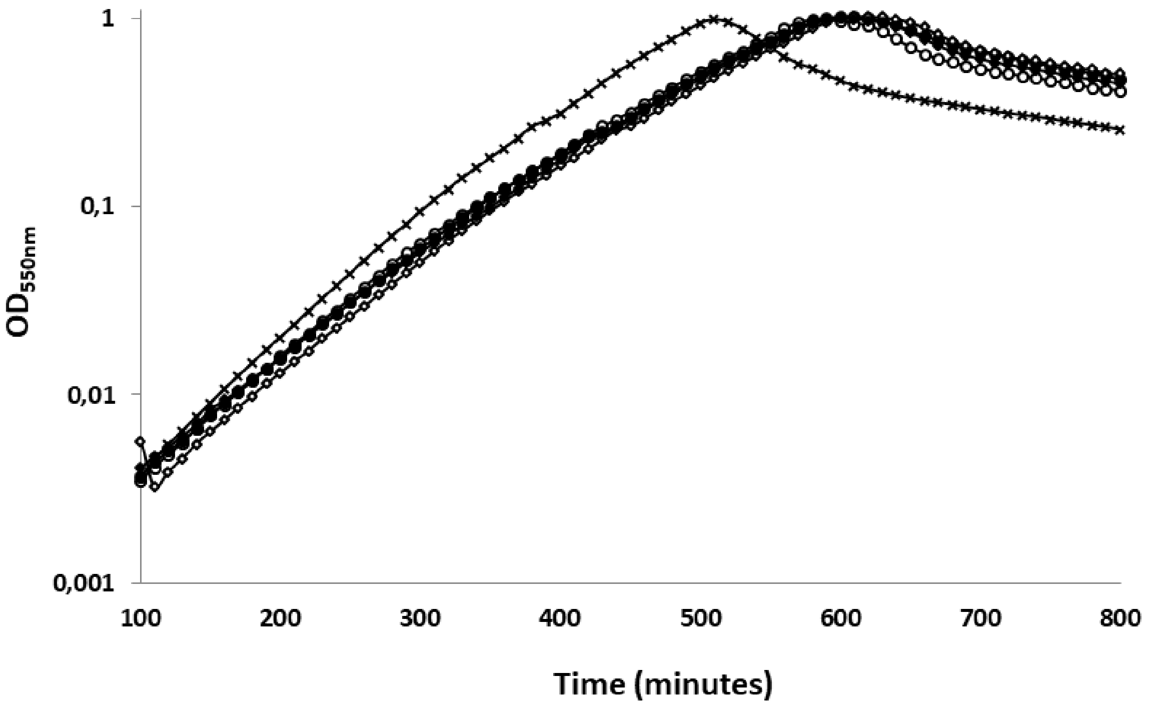

Supplementary figure 3.
Representative growth curves of catalytic / phosphomimetic or –ablative *ubk* mutants
Comparative growth curves at 37°C of the *ubk*-K36R (white circles), *ubk*-Y58E (black circles), *ubk*-Y58F (black crosses), *ubk*-K36R-Y58F (white diamonds) and *ubk*-K36R-Y58E (black diamonds) strains. Bacteria were diluted so that 2.10^5 cells were inoculated at t=0 min. Growths were led in triplicate.

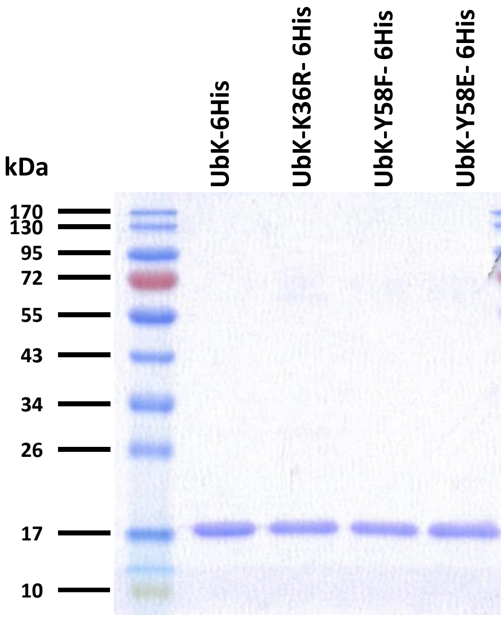

Supplementary figure 4.
SDS-PAGE analysis of purified UbK-6His mutants.
The pneumococcal UbK-6His, UbK-K36R-6His, UbK-Y58F-6His and UbK-Y58E-6His purified from *E. coli* were submitted to gel electrophoresis and stained with Coomassie Blue. 2 µg of each protein were loaded on the gel.

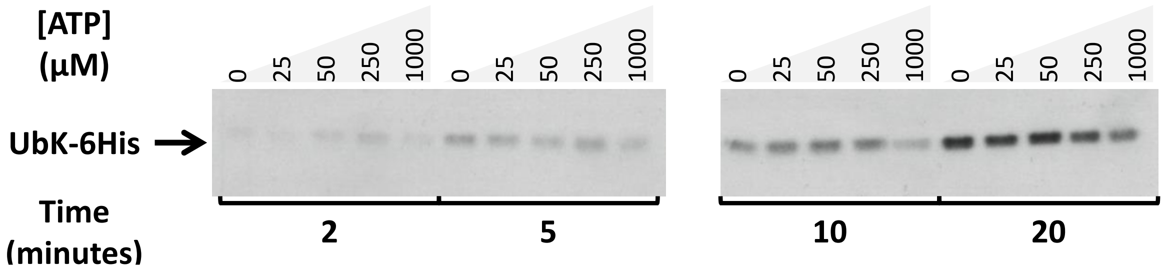

Supplementary figure 5.
Autoradiography of UbK autophosphorylation after different times of incubation and varying concentrations of non-radioactive ATP
UbK-6His purified from *E. coli* was incubated at 37° C during 2 to 20 min with [α-^{32}P]-ATP and varying concentrations of non-radioactive ATP. Mixtures were then analyzed by SDS-PAGE and autoradiography.

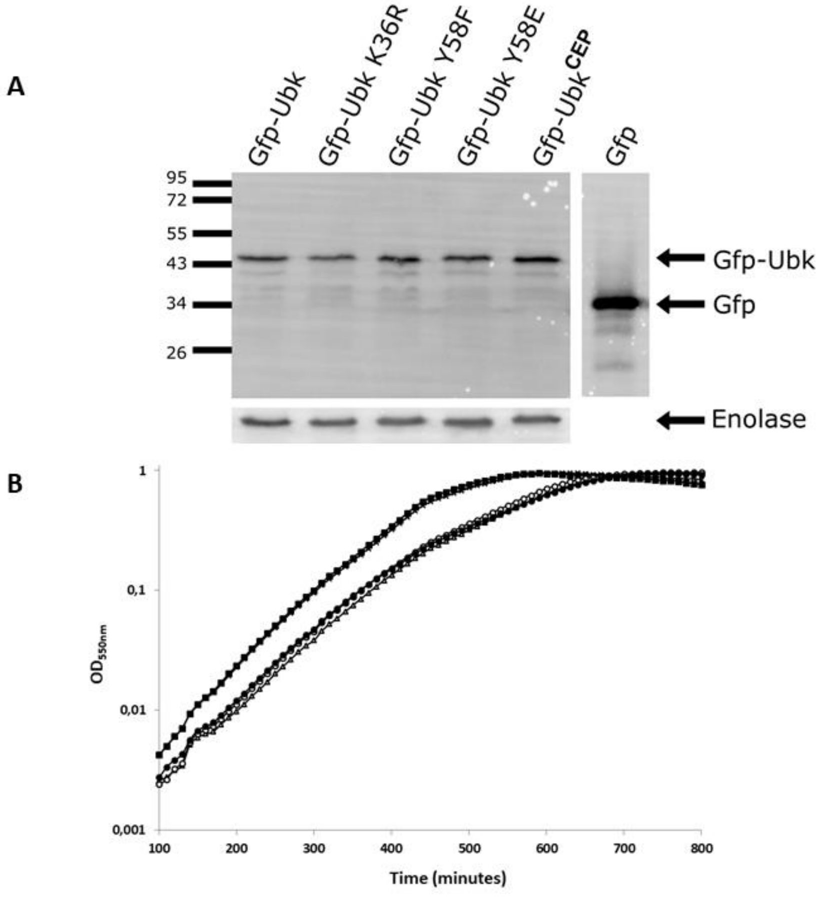

C

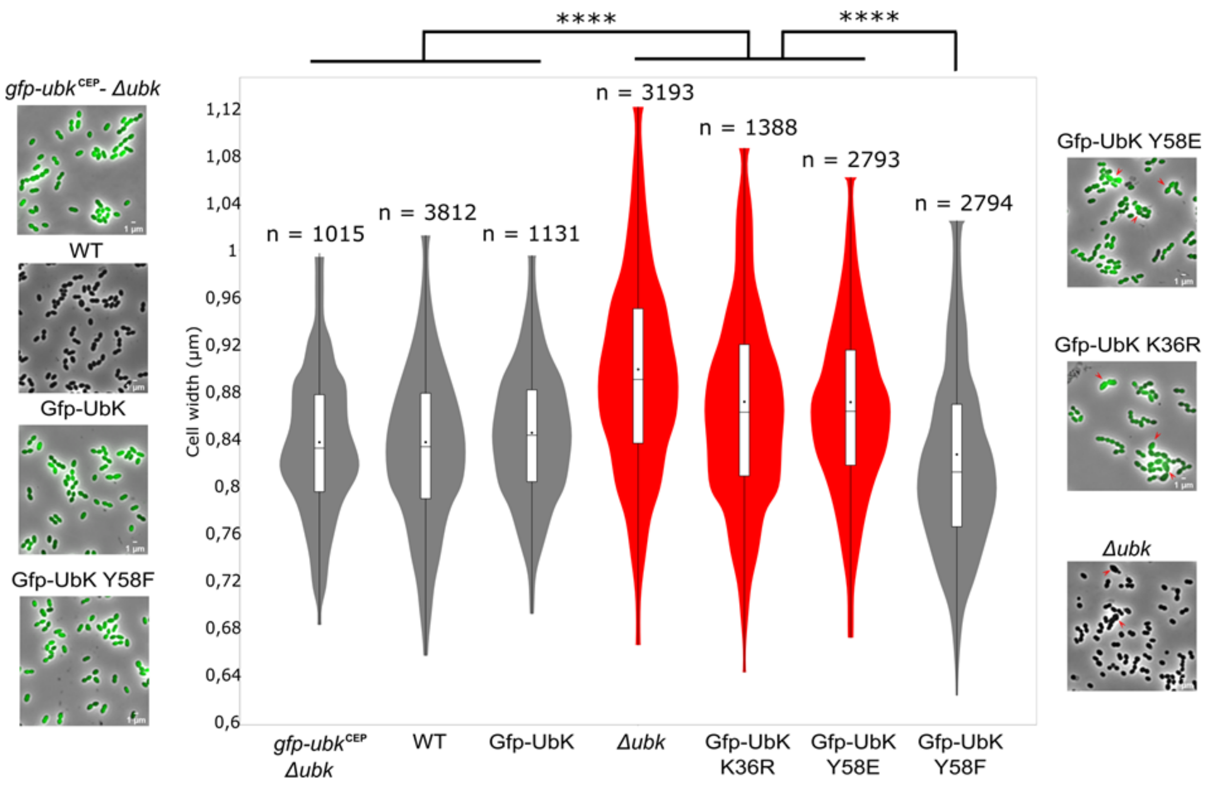

Supplementary figure 6.

Growth curves of the *gfp-ubk* mutants, immunodetection of the GFP-UbK fusion proteins and morphology analysis of *gfp-ubk* mutants

A. Western immunoblot of whole cell lysates from strains *gfp-ubk*, *gfp-ubk*-K36R, *gfp-ubk*-Y58F, *gfp-ubk*-Y58E and *gfp-ubk*^{CEP} and of purified GFP probed with an anti-GFP polyclonal antibody. To estimate the relative quantity of proteins and to compare the different lanes, we used the enolase as an internal standard and detected it with specific antibodies as presented in the lower part of the figure.

B. Representative growth curves at 37°C of the different *gfp-ubk* mutants. Bacteria were diluted so that 2.10^5 cells were inoculated at t=0 min.

Δ*ubk* (white triangles), *gfp-ubk* (black squares), *gfp-ubk*-K36R (white circles), *gfp-ubk*-Y58F (black crosses), *gfp-ubk*-Y58E (black circles).

C. Cell width analysis of *gfp-ubk* mutants. Violin plot showing the distribution of the cell width (µm) measured for each strain. Mann-Whitney test (****P<0.0001). The distribution of the cell width of mutants with morphological defects is shown in red.

The number of cells scored and analyzed is indicated. For each violin, the width of the shaded area represents the proportion of cells located there. The bottom and top of the inside-box represent the 25th and 75th percentile. The bar in
the box indicates the median value while the black dot indicates the mean value.

On phase contrast microscopy images, arrows show swelled mutant cells. Scale bar, 1 µm.

References

Bullock, W.O., Fernandez, J.M., and Short, J.M. (1987). a high efficiency plasmid transforming recA *Escherichia coli* strain with beta-galactosidase selection. *Biotechniques* 5**,** 376.

Cortay, J.C., Negre, D., Scarabel, M., Ramseier, T.M., Vartak, N.B., Reizer, J., Saier, M.H., Jr., and Cozzone, A.J. (1994). In vitro asymmetric binding of the pleiotropic regulatory protein, FruR, to the ace operator controlling glyoxylate shunt enzyme synthesis. *The Journal of biological chemistry* 269**,** 14885-14891.

Fleurie, A., Cluzel, C., Guiral, S., Freton, C., Galisson, F., Zanella-Cleon, I., Di Guilmi, A.M., and Grangeasse, C. (2012). Mutational dissection of the S/T-kinase StkP reveals crucial roles in cell division of Streptococcus pneumoniae. *Molecular microbiology* 83**,** 746-758.

Martin, B., Granadel, C., Campo, N., Henard, V., Prudhomme, M., and Claverys, J.P. (2010). Expression and maintenance of ComD-ComE, the two-component signal-transduction system that controls competence of Streptococcus pneumoniae. *Molecular microbiology* 75**,** 1513-1528.

Studier, F.W., and Moffatt, B.A. (1986). Use of bacteriophage T7 RNA polymerase to direct selective high-level expression of cloned genes. *Journal of molecular biology* 189**,** 113-130.
